# Emergency training in medical practices and resuscitation quality: results from a regional study in Germany

**DOI:** 10.1186/s12873-026-01664-y

**Published:** 2026-07-03

**Authors:** Lorenz Prinz, Vivian Lüdorf, Jan P. Ehlers, Julia Nitsche

**Affiliations:** Didactics and Educational Research in Healthcare, Faculty of Health, Witten/Herdecke University, Alfred-Herrhausen-Straße 50, Witten, 58445 Germany

**Keywords:** Emergency training, Medical practice, Continuing medical education, Resuscitation training, CPR, Feedback

## Abstract

**Background:**

High-quality cardiopulmonary resuscitation (CPR) is essential for improving outcomes in emergency patients treated in medical practices. Although regular emergency training is recommended, its measurable impact on resuscitation quality parameters in this setting has not been sufficiently investigated.

**Objective:**

The aim of this study was to examine the effects of emergency training on the quality of resuscitation measures in medical practices. Specifically, it sought to determine which resuscitation parameters could be influenced by the intervention and whether the quality of care was associated with emergency patient management.

**Methods:**

Seventeen medical practices participated. Each completed a 5-minute resuscitation simulation before (T1) and after (T2) emergency training. Quantitative data were collected on compression rate, compression depth, ventilation frequency, ventilation volume, no-flow time, incorrect hand position and missing defibrillation discharges. Due to small sample size and non-normal distribution, Wilcoxon signed-rank tests were applied. Spearman correlation analysis assessed the association between baseline performance and time since last emergency training.

**Results:**

Significant improvements were observed in ventilation frequency (*p* = 0.007), ventilation volume (*p* < 0.001), ventilation volume per minute (*p* = 0.003), compression depth (*p* < 0.001), and incorrect hand position (*p* = 0.001). Compression rate, no-flow time, and missing discharges did not change significantly. Compression depth increased from 31 [27.5–36.5] mm to 48 [45–56] mm, while ventilation frequency increased from 1.00 [0.00–3.50] to 3.00 [3.00–4.00]. Compression rate remained unchanged (*p* = 0.570). Time since last emergency training correlated with baseline performance (*r*_s_ = −0.516, *p* = 0.034).

**Conclusions:**

Emergency training significantly improves key resuscitation quality parameters in medical practices. Performance declines with increasing time since last training, underscoring the need for regular repetition. Structured, hands-on emergency training appears essential to maintain high-quality CPR performance.

**Clinical trial number:**

Not applicable.

## Introduction

The competencies of German medical practices in managing cardiopulmonary resuscitation (CPR) are considered deficient, and there is a paucity of intensive research in this area [1]. Current studies in Germany primarily assess resuscitation competencies among laypersons [2, 3], whereas competencies among hospital physicians and physicians in outpatient care have not been systematically evaluated in recent years. The most recent available data date back to 2004, when only 20% of hospital physicians were considered competent in providing adequate CPR [4].

Although simulation-based training has been shown to improve emergency skills, its implementation remains underdeveloped in Germany [5]. In 2024, approximately 136,000 individuals experienced an out-of-hospital cardiac arrest in Germany requiring life-saving resuscitation measures [6]. However, it remains unclear how many of these emergencies occur in outpatient medical practices and are managed by practice staff. Furthermore, a structured assessment of the frequency of life-threatening emergencies in outpatient practices is currently lacking. While such events are considered relatively infrequent in primary care, reliable incidence data are unavailable. Existing data sources, including outpatient records, emergency medical services data, and hospital registries, are not systematically linked, limiting comprehensive evaluation of patient pathways and outcomes in relation to outpatient emergency preparedness. Consequently, robust estimates of the true incidence of life-threatening emergencies in outpatient settings and associated patient outcomes cannot currently be derived from available data sources. In addition, emergency teams in Germany frequently lack standardized and comprehensive emergency equipment, which may contribute to suboptimal patient care [7].

Despite the recognized importance of emergency preparedness, resuscitation training in German outpatient practices is not regulated by binding federal law regarding frequency, content, or certification. Requirements are instead embedded in general quality management frameworks, leaving implementation to individual practices [8]. Continuing medical education is therefore largely decentralized and self-regulated. Professional associations, including the German National Association of Statutory Health Insurance Physicians, recommend annual resuscitation training [8], but adherence and structure vary considerably due to the non-binding nature of these recommendations. Overall, the absence of statutory regulation contributes to heterogeneous training practices and variable prioritization in outpatient care.

Internationally, comparable non-binding or indirectly regulated approaches exist, though levels of formalization differ. In the United Kingdom, no statutory requirement defines mandatory resuscitation training frequency in medical practices. The Care Quality Commission (CQC) does not prescribe fixed training requirements [9]; instead, expectations are framed through general quality standards and guidance from bodies such as the Resuscitation Council UK (RCUK) [10]. Under the Health and Social Care Act 2008, providers must ensure staff competence and safe care delivery, which is assessed via CQC inspections.

Similarly, in the United States, CPR training is not federally mandated for outpatient healthcare personnel. The Occupational Safety and Health Administration (OSHA) does not require compulsory CPR certification but emphasizes the need for timely emergency response and recommends appropriate training to ensure immediate first aid capability [11].

In the Asian context, notable differences are observed. In Japan for example, resuscitation training is also not uniformly mandated by law. In contrast, Singapore represents a highly regulated model. All healthcare professionals are required to maintain valid Basic Cardiac Life Support (BCLS) or CPR certification, renewed biennially [12]. The Singapore Resuscitation and First Aid Council (SRFAC), under the Ministry of Health, mandates BCLS training for healthcare personnel, illustrating a structured and enforceable approach to resuscitation training.

Beyond regulatory and structural aspects of resuscitation training, patient outcomes in cardiac arrest are critically determined by the quality and timeliness of CPR delivery. Immediate initiation of chest compressions, maintenance of adequate compression depth and optimal compression rate with minimal interruptions, ensuring adequate ventilation, and prompt defibrillation are critical determinants of patient outcomes [13]. The period during which no effective circulation is established, referred to as no-flow time, must be minimized to preserve artificially maintained circulation generated by chest compressions [14].

Emergency training encompasses a broader spectrum than resuscitation alone. In addition to cardiac arrest management, healthcare professionals are trained in the treatment of conditions such as anaphylaxis, hypoglycemia, and trauma. Common reasons for emergency presentations in outpatient practice include shortness of breath, dizziness, acute abdominal pain, chest pain, and cardiac arrhythmias [1].

A distinctive characteristic of emergency training in outpatient settings is its interdisciplinary structure, in which medical assistants and physicians work collaboratively. No explicit guideline formally regulates this team-based approach. Consequently, European Resuscitation Council (ERC) Basic Life Support (BLS) guidelines [15] are typically supplemented with selected elements from Advanced Life Support (ALS) protocols [7], particularly in team-based training settings.

Experts generally advise against endotracheal intubation by general practitioners, emphasizing non-invasive airway management techniques such as bag-mask ventilation [16, 17]. Accordingly, training focuses on high-quality chest compressions, guideline-compliant ventilation using bag-mask devices, and the establishment of intravenous access where appropriate [7].

Effective emergency management depends on seamless communication and teamwork [18]. Simulation-based training using manikins improves performance and confidence, particularly when combined with structured feedback [19]. Despite technological advances such as virtual reality and artificial intelligence, instructor-led, on-site training remains essential [20].

Routine work in a medical practice requires regular training and continuing medical education (CME) to ensure future quality of care [21]. CME has gained renewed significance during the Coronavirus pandemic [21]. Since 2007, improvements in CME effectiveness have maintained training diversity despite limitations on in-person interaction [21]. Evidence from 34 studies with 35,421 participants indicates that CPR skills deteriorate within 3 months after training, suggesting a negative correlation between time since training and knowledge retention [22]. Physicians who regularly participate in training report greater confidence in performing emergency procedures [6, 23]. The optimal form of emergency training for learning success has not been definitively established, although active participant involvement and tailoring content to the target group improve outcomes [24].

Participation in emergency training has been demonstrated to increase the rate of patients who survive resuscitation [25]. The efficacy of emergency skills training depends on customizing the program to the knowledge level and responsibilities of the interdisciplinary team [18, 26, 27].

The present study aimed to investigate the resuscitation skills of medical practice staff. Specifically, the study examined the effects of an emergency training program on the quality of resuscitation measures in a selection of German medical practices.

### Objectives

1. To determine whether chest compressions and ventilation can be improved through emergency training in medical practice.
2. To establish the optimal frequency of emergency training exercises.

## Methods

### Ethical considerations

The study was approved by the Witten Ethics Committee (application no. S-18/2023).

This study is observational in nature and does not constitute a clinical trial.

### Study design and analysis

This study was designed as an exploratory, hypothesis-generating analysis. The research questions were analyzed quantitatively. Resuscitation parameters from the pre-test (T1) and post-test (T2) were organized in an Excel spreadsheet (version 2309) and analyzed using SPSS (version 29). The results were intended to inform recommendations regarding the optimal frequency of emergency training in medical practices.

### Setting and participants

Practice teams were visited by an instructor for scheduled emergency training sessions. The instructor who conducted the research is a freelancer at Wallmeyer GmbH, a company specializing in emergency training in medical practices. Seventeen medical practices participated in the study between March and June 2023, with each training session conducted in a different practice. Prior to participation, all staff received detailed information about the study, and written informed consent was obtained from all participating individuals.

Medical practices independently contacted Wallmeyer GmbH to arrange emergency training. During the on-site training sessions, L.P. informed practice staff about the study and invited them to participate. Participation was entirely voluntary and had no influence on the delivery of the emergency training. Practices that declined participation still received the full training program as scheduled. In participating practices, two available team members volunteered to take part in the simulation-based assessment and were included in the study. No random sampling or predefined selection criteria were applied. Volunteers were defined as individuals who agreed to participate in both measurement time points (T1 and T2) within the training program.

The practice team included all persons participating in the emergency training, including medical assistants and physicians, with team sizes ranging from four to ten. Participants were required to be of legal age and could withdraw at any time without providing a reason. The professional background of participants was intentionally not collected in detail, as the study was designed to evaluate interprofessional team performance in medical practices without stratification by profession. All participating team members were treated as functionally equivalent members of the resuscitation team within the simulation context. In practice, the participating teams consisted predominantly of medical assistants, with physicians representing a smaller proportion of participants. This distribution reflects the typical staffing structure of primary care practices; however, no formal subgroup analyses were performed based on professional background.

Volunteers were asked to specify the time elapsed since their last emergency training. For practices with two participants, the longer interval since last emergency training was used, assuming that resuscitation performance is constrained by the least recently trained team member. This approach was chosen to reflect the lowest level of recent exposure within the team, which is likely to be most relevant for coordinated emergency performance. All participants reported at least one prior emergency training; therefore, no individuals without previous training were included in the analysis. The time since the last emergency training ranged from 1 to 10 years, with a mean of 3.06 years (*SD* = 2.41). In accordance with data protection regulations, no personal or demographic data were collected.

T1 and T2 assessments were integrated into the scheduled three-hour training program. Most recruited practices were located in North Rhine-Westphalia, with additional practices from Hesse and Rhineland-Palatinate. Medical specialty was not used as a selection criterion; cardiology, gynecology, and general practice clinics were represented. The total number of subjects exceeded the minimum of 10 specified in the ethics application. The limited study duration and research capacity accounted for the reduced sample size. The objective was to recruit two volunteers per practice, aiming for a total of 34 participants.

### Intervention

**Fig. 1:**
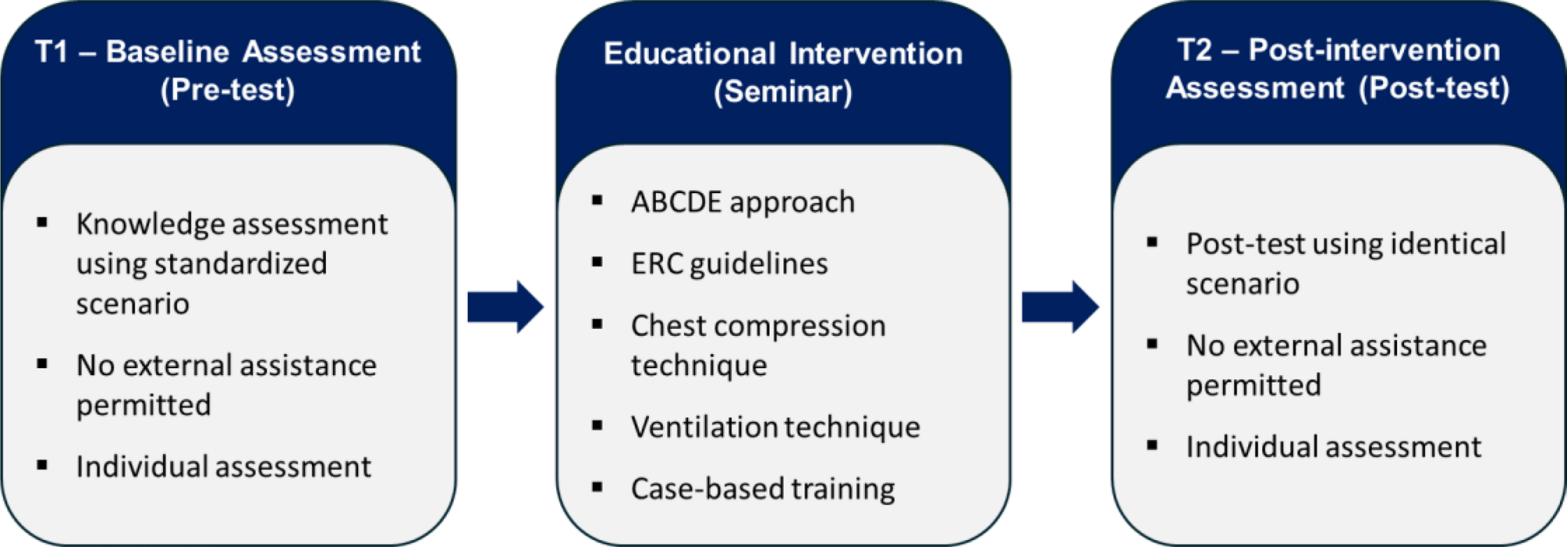
Integration of data collection into the emergency training

The intervention comprised two five-minute resuscitation simulations. As shown in Fig. 1, T1 was conducted prior to the emergency training to assess baseline performance. T2 was conducted after the theoretical and practical segment of the training.

#### Equipment and simulation procedures

The Ambu CPR Software (version 3.1.3) was used to record and display simulations. A resuscitation bag with an appropriate mask and an automated external defibrillator (AED) were provided. Additional materials, including oropharyngeal and laryngeal tubes, a blocker syringe, and a Thomas holder, were available upon request.

During the five-minute simulations, participants received no instructions or feedback. The instructor operated the equipment and distributed materials as requested. All measured parameters were saved locally in PDF format. Recorded parameters included:

- Frequency of ventilation per minute
- Volume of ventilation per attempt
- Volume of ventilation per minute
- Compression frequency per minute
- Compression depth in mm
- No-flow time
- Number of incorrect hand positions
- Number of missing discharges
- Years since last emergency training

Only ventilation attempts resulting in effective ventilation were recorded. Performance was evaluated as team averages rather than by individual participant. Therefore, this study was unable to differentiate between attempts that were not performed and those that were performed unsuccessfully.

The resuscitation parameters were displayed as average values for the five-minute simulation and evaluated as a team performance, rather than being assigned to individual volunteers. The final parameter, designated as “Time since the last emergency training”, was manually incorporated into the PDF document.

#### Training program

Following the completion of T1, the emergency training programme commenced. The theoretical introduction included a discussion of typical emergency scenarios in a medical practice (acute coronary syndrome, anaphylactic reaction after infusion or suspected apoplexy) and the introduction to the ABCDE (airway, breathing, circulation, disability, exposure) scheme, which is used internationally for structured assessment of emergency patients [28]. The guideline-compliant procedure for resuscitation in a medical practice environment was then addressed.

The theoretical component lasted approximately 40 minutes, followed by practical training. Competencies addressed included: head tilt-chin maneuver and evaluation of breathing in unconsciousness patients, chest compression, ventilation of the manikin with bag-valve-mask with face mask, use of the AED, intubation with laryngeal tube and team communication during the resuscitation.

The Ambu CPR software provided personalized feedback by visualizing chest compression depth and ventilation volume. Realistic emergency simulations were conducted subsequently to evaluate the efficacy of the training. Two participants completed the resuscitation simulation under realistic conditions. Participants then exited and re-entered the simulation setting with an unconscious individual (resuscitation manikin). Additional equipment was available, including materials for bag-valve-mask and face mask procedures, intravenous access, sample medication bag administration, oropharyngeal and laryngeal tubes, blocker syringe, stethoscope, AED, and other emergency materials. The emergency simulation was carried out by the same two volunteers from T1 and was used to collect T2 data.

#### Data processing

Data from T1 and T2 were exported from the Ambu CPR software and entered into a spreadsheet using numerical coding, resulting in 17 data sets. Pre-test (T1) and post-test (T2) values were labeled accordingly. Parameter “years since last emergency training” was recorded once per practice and assigned to T1. Data were transferred to IBM SPSS Statistics (version 29) for analysis.

Performance was evaluated using resuscitation parameters defined according to ERC guideline target ranges. Due to the small sample size and non-normal distribution of the data, non-parametric statistical methods were applied. Continuous variables were summarized using median and interquartile range (IQR). Changes between T1 and T2 were analyzed using Wilcoxon signed-rank tests for all resuscitation performance parameters.

To assess the association between training recency and baseline performance, baseline performance was operationalized as chest compression depth at T1. Spearman’s rank correlation coefficient was used to examine the relationship between time since last emergency training and baseline compression depth.

## Results

A total of 17 data sets from T1 and T2 were included in the comparative analysis. Median values and interquartile ranges (IQR) for all parameters are reported in Table 1.

**Table 1:** Comparison of resuscitation performance between T1 and T2 using Wilcoxon signed-rank tests (median and interquartile range)

Parameter	T1 Median [IQR]	T2 Median [IQR]	Z	p	r
Frequency of ventilation per minute	1.00 [0.00–3.50]	3.00 [3.00–4.00]	2.72	0.007	0.66
Volume of ventilation in liters	0.10 [0.00–0.20]	0.30 [0.25–0.40]	3.37	<0.001	0.82
Volume of ventilation per minute in liters	0.10 [0.00–0.55]	1.20 [0.70–1.80]	2.94	0.003	0.71
Compression frequency per minute	118 [99.5–129.5]	109 [101.5–119.5]	−0.57	0.570	−0.14
Depth of compression in mm	31 [27.5–36.5]	48 [45–56]	3.55	<0.001	0.86
No-Flow-Time in seconds	6 [4.5–7]	7 [6–8.5]	1.46	0.145	0.35
Number of incorrect hand positions	5 [0.5–71]	0 [0–0]	−3.18	0.001	−0.77
Number of missing discharges	0 [0–0.5]	0 [0–1]	0.17	0.865	0.04

A significant negative Spearman correlation was observed between time since last emergency training and baseline chest compression depth at T1 (*r*_s_ = −0.516, *p* = 0.034), indicating that longer intervals since the last training were associated with lower compression depth.

## Discussion

The limited number of cases can be attributed to the specific focus of the present study. While comparable literature primarily encompassed individual subjects [29], the present research focused on the team performance of two individuals. Achieving the best possible chances of survival requires at least two people to perform guideline-compliant resuscitation in a well-coordinated manner [7, 30]. Evaluation of team performance is critical in determining the quality of actual resuscitation. This study is distinguished by its unique interprofessional team configuration, which differs from prevailing literature that predominantly compares individuals from the same professions. Integration of diverse professional backgrounds within the practice team facilitates a realistic evaluation of interprofessional team performance.

### Quality of resuscitation measures

The findings indicate substantial enhancement in resuscitation procedures. In our study, pre-existing resuscitation skills among subjects were inadequate. Consistent with previous studies [31, 32], emergency training contributed to improved quality of resuscitation measures. At T1, the ventilation system functioned below the required ERC standard, characterized by inadequate frequency and volume of ventilation. In real-life resuscitation scenarios, such deficiencies could have life-threatening consequences, as reduced blood oxygen levels increase the risk of permanent neurological damage [33]. Chest compressions serve primarily to maintain blood circulation, which is critical for survival, but effective ventilation is equally important for successful resuscitation [34].

No-flow time did not change significantly between T1 and T2 (*p* = 0.145), although a slight increase was observed. This may indicate a potential trade-off between improved ventilation quality and continuous chest compressions, as participants may have spent more time ensuring adequate ventilations during the post-training scenario. However, given the small sample size and lack of statistical significance, this finding should be interpreted cautiously and may also reflect random variation.

#### Quality of ventilation

The quality of ventilation is a key component of high-quality cardiopulmonary resuscitation. Although ventilation is not included in BLS recommendations, it is an essential element in ALS according to ERC guidelines [7, 9]. Ventilation should be performed during pauses between chest compressions at a ratio of 30:2. According to the ERC, ventilation frequency is one of the most significant parameters influencing the outcome of patients with cardiac arrest [7]. At T1, ventilation frequency during bag-mask ventilation was markedly low, with a median of 1 breath per minute (1.00 [0.00–3.50]). Such low ventilation rates may indicate insufficient ventilation delivery during resuscitation scenarios and could potentially compromise oxygenation, as prolonged inadequate ventilation has been associated with adverse neurological outcomes in cardiac arrest patients [33].

The intervention improved ventilation: at T2, ventilation frequency increased substantially compared to baseline (1.00 [0.00–3.50] to 3.00 [3.00–4.00]), although performance remained below ERC-recommended levels. This suggests an improvement in practical skill execution following training, while still indicating the need for continued reinforcement of ventilation competencies [30]. Training also increased delivered ventilation volumes; however, post-intervention values remained below the target range. Despite significant improvements following hands-on training, optimal ventilation performance was not consistently achieved within the five-minute simulation period.

Endotracheal intubation is not recommended in this context of medical practice [35]. A 2018 study found that bag-mask ventilation is not inferior to intubation in out-of-hospital cardiac arrest (OHCA) patients [17]. Supraglottic airway devices are a viable alternative, offering superior airway protection compared with endotracheal intubation in non-hospital settings [36].

#### Quality of chest compressions

During resuscitation, adequate compression frequency and depth are essential [7]. The process depends on consistent and precise execution of mechanical interventions, adhering to high standards [14]. The present study did not show a significant change in compression frequency (T1: 118 [99.5–129.5] vs. T2: 109 [101.5–119.5], *p* = 0.570, *r* = −0.14), but compression depth increased substantially after emergency training (31 [27.5–36.5] mm vs. 48 [45–56] mm, *p* < 0.001, *r* = 0.86), approaching but not reaching the lower limit of the ERC-recommended target range (50–60 mm) [7, 37]. Compression frequency remained within the ERC-recommended range (100–120 compressions per minute) at both T1 and T2. Emergency training also resulted in a significant reduction in incorrect hand positions, from 5 [0.5–71] at T1 to 0 [0–0] at T2 (*p* = 0.001, *r* = −0.77), indicating a large effect of the intervention on correct hand placement during chest compressions.

### Success of the emergency training

Evaluation of T1 results reveals clear deficits due to lack of regular training. Medical practice teams without regular training have insufficient competencies. Chest compression points are often chosen inappropriately, and compression depth is inadequate. Effective ventilation depends on regular practical training to maintain frequency and volume. This finding is based on values from medical practices, most of which had participated in training within the last three years. It is difficult to determine how quality would deteriorate over years or decades without training.

Comparison with T2 results demonstrated the steepness of participants’ learning curves: an approximately three-hour intervention on the premises of the medical practice, including practical exercises on resuscitation manikins, significantly increased the quality of many resuscitation measures. These findings are consistent with the extant literature, which has previously demonstrated the efficacy of resuscitation training with manikins [38] and the positive effect of regular teamwork training on resuscitation quality through brief, repetitive interventions [39]. The importance of regular continuing training to maintain knowledge is well documented [40, 41], although the optimal intervals for repetition have yet to be established. European guidelines recommend a two-year repetition period; in contrast, more frequent refresher intervals have been proposed in the literature, with Anderson et al. suggesting monthly CPR refreshers to optimize skill retention [40].

The temporal relationship between the last emergency training and the quality of resuscitation measures indicates a negative correlation, suggesting that as the interval between training and analysis increases, resuscitation quality declines. This observation is consistent with international literature on CPR skill retention and skill decay following emergency training. Previous studies have demonstrated that resuscitation competencies may deteriorate within only a few months after training, particularly regarding chest compression depth, ventilation quality, adherence to resuscitation algorithms, and team communication [42–44]. Studies therefore emphasize the importance of regular refresher training to maintain both technical and non-technical skills [45]. In particular, brief and repetitive high-frequency training approaches have been associated with improved long-term retention of resuscitation competencies and increased confidence in emergency situations [46, 47].

Although this study did not include clinical outcome data, the findings are limited to simulation-based performance and should be interpreted accordingly. The sample size allowed identification of a trend but was insufficient to determine an optimal repetition interval for refresher training. Taken together with existing guideline recommendations and evidence on skill decay, the results support the relevance of regular refresher training in outpatient medical settings.

### Limitations

This study has several limitations that should be considered when interpreting the findings. First, the sample size was small (*n* = 17), which limits statistical power and reduces the generalizability of the results. Consequently, a priori power analysis was not conducted, and the findings should be interpreted as exploratory and hypothesis-generating rather than confirmatory.

Furthermore, the entirety of the research was undertaken by a single individual. Although a single instructor conducted all sessions at the medical practices, selection of the practices was performed independently by the instructor’s employer, Wallmeyer GmbH. Data collection in the medical practices was voluntary; in the absence of consent, the full emergency seminar occurred without collection of resuscitation data. The specialties of the medical practices were not considered in either the selection or interpretation of the data. Given the limited sample size, this study does not claim full representativeness. Nevertheless, a statistically significant trend was identified, which may be investigated further in future research. Because only two volunteers were recruited per practice team, there is potential for bias from representing a larger team with two participants. In addition, the voluntary participation of particularly well-trained staff may have introduced bias. Data were recorded using the Ambu resuscitation manikin and corresponding software. Despite prior calibration and rigorous checks before each training session, the potential for measurement deviations or inaccuracies due to the sensitivity of the manikin cannot be excluded.

## Conclusions

This study demonstrates that emergency training positively affects the quality of resuscitation measures. Findings showed a statistically significant and substantial improvement in ventilation parameters after emergency training, as indicated by a high observed effect size. Improvements in the efficacy of chest compressions were also substantial. Statistical analysis of the collected data revealed a significant difference in quality across different repetition intervals, indicating that resuscitation quality decreases over time. Further studies are required to determine the optimal interval for repeating emergency training. Preliminary findings suggest that medical practices participate in emergency training too infrequently and irregularly. Practices that engage in regular emergency training demonstrate superior resuscitation knowledge, which may increase the probability of patient survival.

These findings may be relevant beyond the studied outpatient setting and suggest that similar gaps in training frequency and skill retention are likely present in primary care systems internationally. Structured, high-frequency refresher training programs may therefore represent a scalable approach to improving resuscitation performance across comparable healthcare systems worldwide.

## Data Availability

No datasets were generated or analysed during the current study.

## Abbreviations

ABCDE: Airway, Breathing, Circulation, Disability, Exposure Scheme
AED: Automated External Defibrillator
ALS: Advanced Life Support
BCLS: Basic Cardiac Life Support
BLS: Basic Life Support
CME: Continuing Medical Education
CPR: Cardiopulmonary Resuscitation
CQC: Care Quality Commission
ERC: European Resuscitation Council
OHCA: Out-of-Hospital Cardiac Arrest
OSHA: Occupational Safety and Health Administration
RCUK: Resuscitation Council UK
SRFAC: Singapore Resuscitation and First Aid Council
